# Practical Notes and Formulæ

**Published:** 1880-03-20

**Authors:** 


### PRACTICAL NOTES AND FORMULÆ.

**Bedford Iron and Alum Mass.**—Dr. Francis Gillam, of Windsor, North Carolina, writes under date of February 12th, 1880 :

For certain forms of dyspepsia, induced by torpid liver, engorged spleen and nervous prostration incident to malarial poisoning, there is no remedy comparable to Bedford alum and iron mass.

In a case of a lady suffering with anæmia, irritable stomach, intermittent fever, accompanied with a great prostration, it was used with the happiest results. It was with difficulty that this lady retained the simplest food or mildest medicine on her stomach.

For chronic malarial fever, chlorosis, dyspepsia and cachectic condition of the system it is the prince of mineral tonics.

A remarkable cure of chronic diarrhœa and scrofulous affection by this water has recently come under my knowledge.

**Parvules.**—We have found the parvules of Wm. R. Warner & Co. a great convenience in practice. The care with which they are evidently prepared, the certainty and uniformity of effect, and ready solubility of the remedy and dose, and the minute division and neatness of form so convenient to the physician in grading the dose, and so acceptable to the palate of the patient, all combine to recommend this form of medication to the busy practitioner.

Recently, in a case of what we supposed to be *vesicular emphysma*, in a child 14 years old, attended with heart palpitation, dropsical and asthmatic symptoms—a case which had existed for five years and resisted various forms of treatment—we decided to test the effect of digitalis, and prescribed as follows :

℞ Warner's parvules digitalis fol........................ gr. 1–20.

Sig. Take five every night and morning.

The result of this prescription was a cessation to the palpitation, followed by regular diuresis, quiet sleep at night, a return of appetite, and a rapid and remarkable improvement of all the symptoms in the case. So that the little fellow has entirely recovered his health, we believe, permanently, nearly three months having elapsed since a discontinuance of the remedy, and no return of the disease.

The efficiency of these parvules we attribute not to homeopathy but to the thoroughness of preparation and the purity of the articles used. In so far as homeopathy advocates thorough trituration as enhancing solubility and neatness of preparation as more likely to agree with delicate stomachs we make no issue, and we think that often much good results from the avoidance of those disagreeable antipathies which not unfrequently prevents the administration of medicines to delicate persons by reason of the nauseous form in which medicines are ordinarily administered.

**Treatment of Obstinate Eczema.**—In regard to the internal treatment of said disease, says Dr. John S. Aydelotte, in Med. and Surg. Reporter, the only suggestion I have to make, which I think is not generally adopted, is the use of antacids in sufficient doses to establish and maintain a neutral or alkaline condition of the secretions, thereby rendering that of the skin non-irritating, which, I conceive, greatly mitigates the intense itching and burning heat sure to supervene when the patient seeks his couch, gets snuggly wrapped up and in perspiration.

Given in this disease, with other remedies of an essential nature, there need be no fear of any debilitating influence from an alkaline treatment carried to the extent above indicated, and so maintained for an indefinite time.

The following I have found of much service in adult cases :

℞ Sod. bromidi .................................... ℥j.
Sod. bicarbonatis.................................... ʒiiss. M.
Fiat chart. xxx.
Sig.—One powder, in water, a short time before each meal.

℞ Acidi arseniosi.................................... gr. j.
Quiniæ sulphatis....................................
Ferri redacti....................................aa ʒss. M.
Faciat pilulæ xxx.
Sig.—One pill half an hour after each meal.

**Glycerine in Coughs.**—The following are among the formulæ of glycerine and alcohol used by Dr. James in phthisical coughs, etc. Of either of these mixtures, one or two tablespoonfuls may be given three or four times a day :

℞ Glycerine pure....................................f ℥viij.

Spts. frumenti....................................

Tinct. cinchona....................................aa f ℥iv. M.

℞ Vini ferri....................................f ℥iv.

Quinia sulphat.................................... ʒj.

Glycerine pure....................................f ℥viij.

Spts. frumenti....................................f ℥iv.

Dissolve. Useful anti-phthisical remedy with anæmia.

℞ Glycerine pure....................................
Spts. frumenti....................................aa f ℥vj.
Ext. pruni Virginiani fluidi....................................f ℥iv. M.
Useful with a cough, where the tonic and sedative influence of the cherry bark is indicated.

℞ Syrup calcis lacto-phosphate....................................f ℥ij.

Spts. frumenti....................................f ℥viss.

Glycerine pure....................................f ℥vj.

Tinct. cinchona....................................f ℥iss. M.

A very pleasant and useful anti-phthisical prescription.

℞ Salicin.................................... ʒj.

Glycerine pure....................................

Spts. vini Gallici....................................aa f ℥iv.

Dissolve. Useful where a tonic and slight astringent is indicated.

℞ Acidi tannici.................................... grs. xvj.
Glycerine pure....................................
Spts. vini Gallici....................................aa f ℥ij.
Tinct. olei menth. pip.................................... mxvj.
Dissolve. Useful where a more active astringent is required.

℞ Tinct. humuli....................................f ℥iij.

Spts. frumenti....................................f ℥v.

Glycerine pure....................................f ℥viij. M

Useful with a cough, where a tonic is wanted.

**Remedy for Corns.**—Mr. Gezow, a Russian apothecary, recommends the following as a "sure" remedy for corns, stating that it proves effective within a short time, and without causing any pain:

| | | |
|---|---|---|
| ℞ | Salicylic acid | 30 parts. |
| | Extract of cannabis indica | 5 parts. |
| | Collodion | 240 parts. |

To be applied by means of a camel's-hair pencil.

**To Relieve Itching.**—The following is used by Dr. Rigaut, of Paris, in itching from all causes, in lichen, eczema, prurigo, etc., and with very general success wherever the irritation is nervous rather than mechanical:

| | | |
|---|---|---|
| ℞ | Acidi carbolici | gtt. x. |
| | Glycerinæ | ℥ i. |
| | Aquam | ad ℥ i. M. |

To be used in an atomizer, five minutes at a time, several times daily—*Boston Journal of Chemistry*.

**For Asthma, Croup, etc.**—The following is recommended as an inhaling fluid in asthma, croup and kindred complaints:

| | | |
|---|---|---|
| ℞ | Chloroform | 15 parts. |
| | Ether | 30 parts. |
| | Spirits of turpentine | 5 parts. |

Pour a teaspoonful on a cloth, and keep it about three or four inches from the mouth, until the attack is over.—*Boston Journal of Chemistry*.

**Mixture for Acute Gonorrhœa.**—Dr. Jacques Reverdin, of Geneva, prescribes the following mixture at the outset of acute blennorrhagia. It modifies very advantageously the nature of the urine, and is well tolerated by the patient:

| | | |
|---|---|---|
| ℞ | Pulv. sacchari albi | ℥ iij. |
| | Sodii bicarbonatis | ʒ v. |
| | Acidi benzoici, | ʒ iss. |
| | Essentiæ limonis | q. s. |

A teaspoonful to be taken six times a day, in a tumbler of water. To be continued until, the discharge being altered in character, injections and balsams are prescribed.

**For Night Sweats.**—A writer, in Mich. Med. News, says: For over thirty years I have used the following prescription without a single failure in sweats from whatever cause. In one case a neighboring physician was poisoned while dressing a mortified finger. He suffered untold misery and was drenched with perspiration for a number of days, and his life despaired of. When I saw him I ordered him to be bathed immediately and repeat once in two hours. The third application stopped all perspiration, and convalescence commenced at once:

| | | |
|---|---|---|
| ℞ | Alcohol | Oj. |
| | Sulphate of quinine | ʒj. M. |

Wet a small sponge with it and bathe the body and limbs, a small surface at a time, care being taken not to expose the body to a draft of air in doing it.

**Quinine in Whooping Cough.**—A British authority recommends the following as "almost a specific" in this disease:

℞ Quiniæ sulphatis.................................................... ℈ j.

Sol. acid hydrobromic (Fothergill)........................ ʒ iss.

Syrupi altheæ.................................................... ℥ iss.

Aquæ.......................................................ad ℥ vi.M.

A dessertspoonful 4 times a day; the dose to be increased according to age.—*Med. and Surg. Reporter.*

**Recent Suggestion for Ozena.**—To remove the crusts, Dr. Lennox Browne, in Medical Press and Circular, uses:

℞ Iodoformi.................................................. gr. vi–viij.

Ætheris ....................................................... ʒ j–iss.

Ung. petrolei................................................ ℥ j.

Ottar rosæ.................................................... mvj.

Dissolve the iodoform in the ether, then add the others.

For a post-nasal douche:

℞ Ammonii chloridi.............................................

Sodii boratis.........................................aa gr.vi–viij.

Glycerinæ.................................................... ʒ j–ij.

Aquam...............................................ad ʒ iv. M.

This amount for two douches, at 95° Fah.

For vapor inhalations, either pine oil, creosote, or benzole, in water, at 150° Fah. should be inspired by nose as well as by throat. To whichever is prescribed, aldehyde, in no larger proportion than one drop to each inhalation, should be added, this drug having a peculiar and quite specific effect on favoring fluid secretions in cases of inspissated mucus, and if administered in large doses, it is apt to produce headache or embarrassment of breathing.

In the British Medical Journal, he gives other formulæ:

℞ Sodii boratis.............................................. ʒ iij.

Acidi salicylici............................................ ʒ ij.

Glycerinæ.................................................. ℥ ijss.

Aquam ...............................................ad ℥ iij.

One or two drachms of this mixture to the half pint of water, at 95° Fah., acted quite efficiently, whether used with anterior or post-nasal douche, or as a gargle; and this form has now been used by him for many months. It has the advantage, over and above its antiseptic qualities, of being not only non-irritating, nor obnoxious in taste, but, on the contrary, of being even emollient, and of agreeable flavor —*Med. and Surg. Reporter.*

**Nervous Debility, Dyspepsia, etc.**—Dr. Lindsay Johnson, of Atlanta, furnished us the following:

℞ Elixir colisoya iron and strychnia.................... ʒ iij.

Sig. Teaspoonful after each meal.

The above formula, prepared by R. A. Robinson & Co., of Louisville, Ky., is admirably adapted to all affections denoting enervation,

